# Current status and related factors of turnover intention of primary medical staff in Anhui Province, China: a cross-sectional study

**DOI:** 10.1186/s12960-021-00563-6

**Published:** 2021-02-27

**Authors:** Guimei Chen, Lingzhi Sang, Jian Rong, Huosheng Yan, Hongzhang Liu, Jing Cheng, Li Wang, Hong Ding, Ren Chen

**Affiliations:** School of Health Management, Anhui Medical University, No. 81, Meishan Road Shushan District, Hefei, 230032 Anhui China

**Keywords:** Primary medical staff, Turnover intention, Psychological capital, Social support, Job burnout

## Abstract

**Background:**

The shortage of primary medical staff is an important issue in the management of health human resources, and it is also a problem that all countries in the world need to face together. Since 2009, China has implemented a new series of medical system reforms and the shortage and loss of primary medical staff have been alleviated accordingly. However, China has a large population and it is difficult to distribute health human resources evenly across regions. This study aimed to explore the current status of turnover intention and its relationship with psychological capital, social support, and job burnout, as well as how these factors influence turnover intention of primary medical staff in Anhui province, China.

**Methods:**

Using structured questionnaires to collect data, including demographic characteristics, turnover intention, psychological capital, social support, and Chinese Maslach Burnout Inventory scale. A total of 1152 primary medical workers of Anhui were investigated. Data were analyzed by *t*-test, analysis of variance (ANOVA), Pearson correlation analysis, and multiple linear regression model.

**Results:**

Total scores of turnover intention, psychological capital, social support, and job burnout of subjects were 14.15 ± 4.35, 100.09 ± 15.98, 64.93 ± 13.23 and 41.07 ± 9.437, respectively. Multiple linear regression showed the related factors of turnover intention were age, job position, work unit, and scores of job burnout. Pearson correlation showed psychological capital and social support were negatively correlated with turnover intention, while the score of job burnout was positively correlated with turnover intention.

**Conclusion:**

The improvement of psychological capital and social support and the reduction of job burnout may play an important role in reducing turnover intention of primary medical staff. Primary medical managers should strengthen the humanistic care for primary medical staff, optimize the incentive mechanism, and improve internal management of medical institutions for stability.

## Background

Due to China’s large population, there is a large demand for health human resources. However, the shortage of primary medical staff is a leading problem. Primary medical staff are the direct care providers, an irreplaceable role in popularizing medical and health knowledge and improving their self-health promotion level. Therefore, “without the labor force in health care, there is no health” is a generally accepted theory [1]. The *China Statistical Yearbook 2019* showed the total number of health workers in China was 12.301 million, of which 3.826 million were primary health workers (only 31.1%): 14.5% in community health service centers (stations), 35.5% in township health centers, 29.9% in village clinics, and 20.1% in other primary healthcare institutions (including street health centers, outpatient departments and clinics) [2].

With the outbreak of the coronavirus pandemic, a Chinese scholar proposed implementing various prevention and control measures in the community [3]. This pandemic highlights the necessity of primary medical staff; however, it also creates problems for them like increased workload, work pressure and job burnout, and reduced job satisfaction, having profound effects on job satisfaction and enthusiasm. Adding these problems to the lacking incentive mechanism, there is bound to be a serious impact on stability of primary medical staff, all of which also explain why most Chinese medical students rush to work in city hospitals after graduation [4]. In 2015, Meng et al. [5] stated that about 8% of primary healthcare workers left their hospitals, and half switched to higher-level hospitals (such as the second-class and third-class hospitals). Moreover, the World Health Organization (WHO) announced the shortage of primary medical staff is a global problem [6]. During the Third Global Forum on Human Resources for Health, WHO revealed that by 2035, the world will have 12.9 million less medical staff members. Under this circumstance, WHO has proposed a series of evidence-based global policy recommendations promoting the enthusiasm of medical staff in remote and rural areas by improving working conditions and creating incentives [7].

Anhui is a typical region for primary healthcare reform in China. It has always been committed to improving the social medical insurance system, reforming the public hospital system and strengthening primary healthcare in rural regions [8]. However, the basic health human resources in Anhui are relatively scarce, and the gap between urban and rural areas is vast; in 2019 there were 2.01 practicing physicians and practicing assistant physicians per 1000 people, of which 2.69 were urban and 1.39 were rural. There are 2.37 registered nurses per 1000 people, of which 3.83 are urban and 1.35 are rural. There are 5.27 health technicians per 1000 population, of which 7.68 are urban and 3.39 are rural [2]. Anhui’s population accounts for 4.5% of China, while the medical staff accounts for only 1.8% (224,000) and the primary medical staff accounts for 3.5% (135,000) of the country.

Psychological capital is a positive psychological force generated by a person’s positive emotional state to individual development and organizational construction and is an important source of organizational competitiveness [9]. It is a kind of capital other than human capital, social capital and financial capital which can surpass human and social capital and promote the sustainable development of individuals [10]. Social support is the personally perceived support of individuals, emphasizing the individual’s self-understanding, experience and feelings about social support from different sources [11]. Job burnout is a state in which a person suffers from physical and mental fatigue due to excessive work stress beyond his or her own scope [12]. Turnover intention is the tendency to leave the current job to find a new one. Chinese and international studies have shown that an important ideological act and premise leads to departure behaviors and has a good predictive power for actual departure behaviors [13].

Our study focused on primary medical staff in Anhui to understand the status of their turnover intention, analyze the influencing factors of their turnover intention, and explore the relationship between psychological capital, social support, and job burnout and their turnover intention. The results of this study will provide a reference for stabilizing the primary medical team, improving quality of primary medical services, and formulating management strategies for primary medical staff.

## Methods

### Studying setting

Anhui is located east of central China, and its economic development is at a medium level across the country. Each year, 22,635 primary health institutions in Anhui provide outpatient and inpatient services, accounting for 61.27% and 20.90%, respectively, which are higher than the national average of 54.12% and 18.21% [14].

This province was selected for the following reasons: (1) Anhui has been at the forefront of China in the comprehensive reform of primary medical institutions making its primary healthcare institutions a government priority. (2) Considering feasibility, Anhui guaranteed the compliance of participants. (3) Anhui has a large number of primary healthcare workers.

### Participants and data collection

We used a cross-sectional survey design. Primary medical staff in this study refers to the medical staff in community health service centers (CHC), community health service stations (CHS), township health centers, village clinics, and outpatient departments. According to the regional characteristics, Anhui is divided into three regions: Northern Anhui, Central Anhui, and Southern Anhui. We used random sampling to select a district and a county from southern and central Anhui and one district and two counties from northern Anhui (northern Anhui has a larger population). We used a series of questionnaires to collect data, and the respondents filled out the questionnaires anonymously and voluntarily. The questionnaires in each area were collected by special investigators and withdrawn immediately after completion. If the respondent answered regularly to the questionnaires or filled in the questionnaires with incomplete content, it would be regarded as invalid and eliminated.

Before conducting the survey, we held several investigator trainings and set inclusion and exclusion criteria (survey subject must be over 20 years old, held their position for one year, occupation of medical staff is limited to doctors and nurses, pharmacists, administrative staff, etc.). We also held a series of on-site, preliminary investigations before official investigation.

We recovered 1300 questionnaires, of which 1152 were valid, making the effective recovery rate 88.62% (1152/1300).

## Measures

### Demographic characteristics questionnaire

First, we used the general characteristics questionnaire, which we independently designed based on relevant literature and expert consultation, including three parts: (1) general demographic characteristics: gender (male, female), age (20–30 years old, 31–40 years old, 41–50 years old, over 50 years old), professional title (primary, intermediate and above), education (secondary; technical school and below, college; undergraduate and above), working years (1–10 years, 11–20 years, 20 years or more), marital status (married, other). (2) Job characteristics: monthly income (CNY 3000 and below, higher than CNY 3000), occupations (doctor, nurse, pharmacist and administrative staff), work unit (CHC, CHS, township health center, village clinic, outpatient department) (3) Regional characteristics: The city or county (district) where the respondents are located.

### PCQ-24

Psychological capital is measured using the Psychological Capital Scale (PCQ-24) compiled by Luthans et al. [15], with a total of 24 items. It measures psychological capital from four dimensions: self-efficacy, hope, resilience, and optimism. Points are graded from 1 to 7. A score of 124 indicates extremely high psychological capital; above 100, is high level of psychological capital; above 80, the psychological capital is a medium level; below 80, it is necessary to strengthen and train psychological capital. The reliability of the scale is Cronbach's α coefficient of 0.97, and the results of confirmatory factor analysis showed the scale has good reliability.

### PSSS

We used *Perceived Social Support Scale (PSSS)* compiled by Zaimet et al. [16] in 1987 and revised by Chinese scholar Wang XD [17]. The scale consists of 12 items, divided into two dimensions: in-family support (items 3, 4, 8, 11), and out-of-family support (the remaining items). Points 1 to 7 are used for scoring. Scores between 12 and 36 are considered low support state; between 37 and 60 points are an intermediate support state; between 61 and 84 points are a high support state. The reliability of the scale is Cronbach’s α coefficient is 0.94.

### Job burnout scale

We used the Chinese Maslach Burnout Inventory (CMBI) of Li et al. [18] for scoring. It is revised based on MBI (Maslach Burnout Inventory) questionnaire [19]. There are 15 questions in the questionnaire, with five questions in each three dimensions: emotional exhaustion, disintegration of personality, and reduction in sense of achievement. It uses Likert's 7-point scoring method, 1 means “strongly disagree” and 7 means “strongly agree”. The scale divides the level of burnout into four levels through three critical values: 25 points for emotional exhaustion, 11 points for disintegration of personality, and 16 points for reduced sense of achievement. When all three dimensions are less than the critical value, it is zero burnout; when any of the three dimensions is higher than the critical value, it is mild burnout; otherwise, it is moderate or severe burnout. The internal consistency test of the scale showed a Cronbach’s α coefficient of 0.767, indicating good reliability.

### Turnover intention scale

The scale of turnover intention was translated and revised by Michael et al. [20]. The scale includes a total of six items, divided into three dimensions: possibility of quitting current job (turnover intention I, items 1 and 6), motivation to find other jobs (turnover intention II, items 2 and 3), and obtaining external possibility of work (turnover intention III, items 4 and 5). It uses reverse scoring on a scale of 1 to 4. If the score is higher, the turnover intention is higher. The reliability of the scale is Cronbach's α coefficient of 0.80.

### Statistical analyses

We used Epi Data 3.1 for database building, and researchers double-entered data and performed error detection, SPSS 20.0 (IBM Corp, Armonk, NY, USA) for statistical analysis of data. Count data are described by composition ratio and measurement data are described by mean and standard deviation (*M* ± SD).

For univariate analysis, we conducted an independent sample *t*-test for binary variables (gender, professional titles, marital status, monthly income). Then, we divided the age of primary medical staff into four groups: 20–30 years old, 31–40 years old, 41–50 years old, 50 years and older; divided the working years into three groups: 1–10 years, 10–20 years, more than 20 years, which changes the age and working years of primary medical staff from continuous variables to categorical variables. We performed one-way analysis of variance (ANOVA) on multiple categorical variables (age, education, working years, employment agency, occupation, region). In addition, we used Pearson correlation analysis to explore the correlation between turnover intention and the scores of psychological capital, social support, and job burnout among them, the test level *α* = 0.05.

In multivariate analysis, we used multiple linear regression models to set dummy variables for categorical variables and set a control for each survey item, thereby testing its association with other items under standard and non-standard coefficients, the test level *α* = 0.05.

## Results

### Demographic characteristics of participants

We surveyed 1152 people, including 520 males and 632 females. The age group was mainly distributed between ages 31–40 and 41–50, with an average age of 40.17 ± 8.46. Their professional titles were mostly junior titles (80.4%); marital status was mostly married (91.4%); educational backgrounds were mainly secondary and below and junior colleges, accounting for 43.4% and 39.4%, respectively. The average working years was 18.14 ± 8.91 years; the number of people with an average monthly income of < RMB 3000 and ≥ RMB 3000 was 565 and 587, respectively. Among them, physicians, pharmacists, nurses, and administrative staff were 602, 74, 243, and 233, respectively. Of the primary medical staff surveyed, 714 worked in township. In Table 1, the *t*-test and analysis of variance (ANOVA) were used to test the univariate variables.

**Table 1:** Demographic characteristics of participants (n = 1152)

Demographic variable	Composition ratio (%)	Scores of turnover intention $\left( {\overline{x} \pm {\text{s}}} \right)$	*t*/*F*	*P-*value
Sum	1152 (100.0)	14.24 ± 4.33	–	–
Gender			6.88	0.009*
Male	520 (45.1)	14.61 ± 4.62		
Female	632 (54.9)	13.93 ± 4.06		
Age (years)			11.78	< 0.001**
20–30	181 (15.7)	13.28 ± 3.92		
31–40	373 (32.4)	14.94 ± 4.22		
41–50	497 (43.1)	14.36 ± 4.50		
≥ 50	101 (8.8)	12.75 ± 4.01		
Professional titles			0.01	0.922
Junior title	926 (80.4)	14.23 ± 4.39		
Middle title and above	226 (19.6)	14.26 ± 4.08		
Education level			2.47	0.085
Secondary school and below	500 (43.4)	14.49 ± 4.49		
Associate degree	454 (39.4)	13.89 ± 4.26		
Bachelor degree and above	198 (17.2)	14.41 ± 4.05		
Working years (year)			6.63	0.001*
1–10	293 (25.4)	13.96 ± 4.24		
11–20	412 (35.8)	14.85 ± 4.34		
≥ 21	447 (38.8)	13.85 ± 4.33		
Marital status			6.33	0.012*
Married	1053 (91.4)	14.34 ± 4.35		
Unmarried	99 (8.6)	13.19 ± 4.01		
Monthly income (CNY)			0.86	0.354
< 3000	565 (49.0)	14.36 ± 4.39		
≥ 3000	587 (51.0)	14.12 ± 4.28		
Occupation			15.36	< 0.001**
Physicians	602 (52.3)	15.03 ± 4.45		
Pharmacists	74 (6.4)	13.85 ± 4.03		
Nurses	243 (21.1)	13.32 ± 3.73		
Medical managers	233 (20.2)	13.26 ± 4.30		
Work units			8.72	< 0.001**
CHC	126 (10.9)	13.29 ± 4.45		
CHS	46 (4.0)	14.37 ± 3.96		
Township hospitals	714 (62.0)	13.92 ± 4.20		
Village clinics	179 (15.5)	15.69 ± 4.56		
Outpatient department	87 (7.6)	15.15 ± 4.19		
Regions			10.89	< 0.001**
North of Anhui	804 (69.8)	14.00 ± 4.36		
Middle of Anhui	210 (18.2)	15.47 ± 4.30		
South of Anhui	138 (12.0)	13.77 ± 3.89		

*CHC* Community Health Service Center, *CHS* Community Health Service Station

* *P* < 0.05, ** *P* < 0.001

### Description of psychological capital, social support, job burnout and turnover intention

In Table 2, the total psychological capital score was 100.07 ± 15.90 points, of which the self-efficacy score was 24.96 ± 4.48, the hope score was 24.70 ± 4.41, the resilience score was 25.16 ± 4.40, and the optimistic score was 25.24 ± 4.48 min. The social support score was 64.98 ± 13.25 points, including family support 22.53 ± 4.94, friend support 21.42 ± 4.79, and other support points 21.04 ± 4.83. The job burnout score was 41.07 ± 9.437 points, of which emotional exhaustion was 13.83 ± 5.018, work attitude was 9.34 ± 4.684, and low sense of achievement was 17.90 ± 4.424. The total score of turnover intention was 14.15 ± 4.35, including turnover intention I 5.26 ± 1.50, turnover intention II 4.04 ± 1.80, and turnover intention III 4.85 ± 1.89.

**Table 2:** Scores of psychological capital, social support, job burnout and turnover intention

Items	M ± SD
Total score of psychological capital	100.07 ± 15.90
Self-efficacy	24.96 ± 4.48
Hope	24.70 ± 4.41
Toughness	25.16 ± 4.40
Optimistic	25.24 ± 4.48
Total score of perceived social support	64.98 ± 13.25
Family support	22.53 ± 4.94
Friends support	21.42 ± 4.79
Other supports	21.04 ± 4.83
Total score of job burnout	41.07 ± 9.437
Emotional exhaustion	13.83 ± 5.018
Working attitude	9.34 ± 4.684
Low sense of achievement	17.90 ± 4.424
Total score of turnover intention	14.15 ± 4.35
Turnover intention I	5.26 ± 1.50
Turnover intention II	4.04 ± 1.80
Turnover intention III	4.85 ± 1.89

### Correlation analysis

In Table 3, we used Pearson correlation analysis to explore correlation between turnover intentions and psychological capital, social support, and job burnout. The results showed a negative correlation between turnover intentions and psychological capital and social support (*p* < 0.05) and a positive correlation with job burnout (*p* < 0.05).

**Table 3:** Results of correlation analysis of turnover intention

Project	Turnover intention	
	*r*	*p-*value
Psychological capital	− 0.079	0.006
Social support	− 0.085	0.003
Job burnout	0.129	< 0.001

### Regression analysis

In Table 4, The score of intention to leave was the dependent variable, while gender, age, professional title, education background, working years, marital status, monthly income, occupation, work unit, region, scores of psychological capital, social support, and job burnout were independent variables. The results of multiple linear regression analysis showed age, occupation, work unit, and scores of job burnout were influential factors of turnover intention *(p* < 0.05). In different occupations, doctors' turnover intention was higher than that of nurses (*B* = − 1.557) and medical managers (*B* = − 1.490); there were also differences in different work units: the table shows that staff of village clinics (*B* = 1.284) and staff of the outpatient department (*B* = 1.4457) had higher turnover intention than those working at community health service centers. Job burnout was another factor influencing willingness to leave (*p* = 0.001). Results show correlation indicating that the stronger the job burnout, the higher the turnover intention.

**Table 4:** Multiple linear regression model for turnover intention

	Unstandardized coefficients		Standardized coefficients	*t*	*p-*value	95.0% CI
	*B*	SE	*β*			
Constant	17.384	1.808		9.613	< 0.001	(13.836, 20.932)
Gender (male^a^)						
Female	0.214	0.303	0.025	0.707	0.480	(− 0.381, 0.809)
Age (years)	− 0.091	0.035	− 0.177	− 2.562	0.011*	(− 0.16, − 0.021)
Professional titles (junior title^a^)						
Middle title and above	0.374	0.345	0.034	1.083	0.279	(− 0.303, 1.051)
Education level (secondary school and below^a^)						
Associate degree	− 0.194	0.301	− 0.022	− 0.643	0.520	(− 0.784, 0.397)
Bachelor degree and above	0.176	0.402	0.015	0.439	0.661	(− 0.612, 0.964)
Working years	0.038	0.033	0.077	1.141	0.254	(− 0.027, 0.102)
Marital status (married^a^)						
Unmarried	− 0.949	0.484	− 0.061	− 1.963	0.050	(− 1.899, 0.000)
Monthly income (< 3000^a^)						
≥ 3000	− 0.296	0.270	− 0.034	− 1.098	0.272	(− 0.826, 0.233)
Occupation (physicians^a^)						
Pharmacists	− 0.949	0.544	− 0.054	− 1.746	0.081	(− 2.016, 0.118)
Nurses	− 1.557	0.400	− 0.147	− 3.892	< 0.001**	(− 2.343, − 0.772)
Medical managers	− 1.490	0.356	− 0.138	− 4.191	< 0.001**	(− 2.187, − 0.792)
Work units (CHC^a^)						
CHS	0.843	0.731	0.038	1.153	0.249	(− 0.591, 2.278)
Township hospital	0.560	0.466	0.063	1.203	0.229	(− 0.353, 1.474)
Village clinics	1.284	0.560	0.107	2.291	0.022*	(0.184, 2.383)
Outpatient department	1.445	0.621	0.088	2.328	0.020*	(0.227, 2.663)
Regions						
North of Anhui	0.652	0.368	0.058	1.772	0.077	(− 0.070, 1.374)
Central of Anhui	− 0.242	0.469	− 0.018	− 0.516	0.606	(− 1.162, 0.678)
Psychological capital	− 0.007	0.009	− 0.026	− 0.759	0.448	(− 0.025, 0.011)
Social support	− 0.009	0.011	− 0.028	− 0.834	0.405	(− 0.031, 0.012)
Job burnout	0.043	0.013	0.094	3.206	0.001*	(0.017, 0.069)

^a^Constant

* *P* < 0.05, ** *P* < 0.001

## Discussion

### Status of turnover intention of primary medical staff in Anhui

In recent years, Anhui has made achievements in strengthening the construction of primary medical staff. Compared with the turnover intention of rural primary medical staff in Guizhou Province [21], the turnover intention of primary medical staff in Anhui is relatively low. Nevertheless, the data of this study indicate the primary medical staff in Anhui still has risk of leaving, and a high turnover intention. The scores of each dimension (possibility to quit current job, motivation to find other jobs, possibility to obtain external jobs) are also high. This depicts turnover intention and risk of leaving for other jobs has reached a relative high level, similar to the findings in a research report on turnover intention of primary-level medical staff in multiple Chinese provinces, demonstrating the tendency of this problem across China [22]. The results of this study provide baseline information for researchers in other Chinese provinces.

Compared with a study from Iraq [23], our respondents have higher turnover intention, possibly due to better infrastructure of the country's primary medical institutions and compensation for medical staff, making them more satisfied with their jobs. One UK report [24] revealed a survey of 1192 community GPs (general practitioners) in England where 41.9% planned to leave their current jobs, demonstrating that other nations also have problems with high turnover intention of foreign primary medical staff. Researchers need to consider the results of these studies comprehensively, find commonalities between them, and consider differences between countries to propose effective solutions.

### Influencing factors of turnover intention

In this study, turnover intention score was the dependent variable, and demographic variables, job characteristics, regional characteristics, scores of psychological capital, social support, and job burnout were the independent variables. The results of multiple linear regression analysis showed that age, occupations, work units, and scores of job burnout were the influencing factors of turnover intention (*p* < 0.05).

### Age (years)

This study found differences in the turnover intention scores among different age groups: middle-aged people (31–50) have the highest scores, while scores of young people (30 and below) and older people (over 50) are relatively low, which is inconsistent with other previous studies [25], showing young doctors are more likely to leave. Different findings can be attributed to status of primary healthcare systems in different countries; in China, young medical staff often lack business experience and social relationships, so middle-aged medical staff may have superior medical skills and richer social relationships [26]. This makes them more willing to seek higher income and social status and more likely to consider leaving existing posts. In addition, the majority of Chinese nursing staff is young people just as the primary nursing staff in Anhui (42.8% of the young people in this study data). The main force of primary healthcare workers are young, energetic nursing staff, with less family burden, allowing them to devote themselves to heavy work more fully. Moreover, nursing work is often the key to treatment and rehabilitation, making it necessary to pay attention to young medical staff at the grassroots level.

### Occupations

This study finds that doctors have different turnover intention scores when compared to medical staff in other positions (nurses, pharmacists, etc.). At present, there are many studies on turnover intention of nursing staff at home and abroad, like Heinen’s [27], whose findings showed that 9% of nurses intend to leave their profession, and the ratio varies from 5 to 17% across the European countries. Our research found doctors have higher scores of turnover intentions than other positions, contrary to the findings of Sachiko Minamizono [28] and Van der Heijden [29], who reported nurses have higher willingness to turnover than doctors.

Among primary doctors we surveyed, less than 30% completed the bachelor’s degree. Nonetheless, most newly recruited doctors must receive the standardized residents training in hospital and other clinical skills assessments. In such a long training process, professional skills increase rapidly. However, almost all nurses will enter medical institutions after completing their bachelor or associate degree, and most will no longer receive professional skills training and will only gradually enhance nursing skills in daily work. Our study also showed more than 50% of primary nurses have an associate degree but less than 20% have a bachelor's degree, and the proportion of primary doctors with a bachelor's degree is higher than that of nursing staff. In addition, the number of primary doctors in the study group aged 31–50 was more than nurses of the same age group, which is similar to the age distribution in another study on primary medical staff in China [22], providing further evidence that medical staff in this age group are more willing to leave than others. Doctors with more personal ability are full of expectations for future development and consider the possibility of finding external work more often, they bear the dual pressures of department work and non-medical work, and do not rule out the possibility of burnout and turnover. It also confirms why doctor's turnover intention score is higher than nurses and other occupations.

According to medical ethics, traditional Chinese gender roles define men as being more achievement oriented and adventurous than women [30]. Forced by social and familial responsibilities, men think more about changing career plans. In China, the majority of nurses are female; therefore, we can conclude nurses are less likely to leave their jobs than men, as most nurses are women and most doctors are men.

### Work units

Medical staff in different medical institutions also have different turnover intention scores. Medical staff in village clinics have the highest scores, while community medical service stations (CHS) and service centers (CHC) are lower. It is similar to the results of a Chinese scholar's research on the current situation of village-level medical institutions and personnel in Anhui [31]. Therefore, China’s primary medical service system is not yet complete, and village-level health institutions and health personnel face large difficulties.

### Job burnout

Finally, medical staff job burnout also needs attention. One Chinese study [32] found that 68.42% of general practitioners in primary healthcare institutions in the province work more than 8 h a day and 44.65% work more than 6 days per week; more than 60% general practitioners believe that the content of current work is cumbersome; and most young doctors who just started working have difficulty adapting to their current work; 67.21% of them need to take on other non-medical work. Consequently, medical workers often feel tired from work and do not rule out leaving their current jobs. A foreign scholar has also clearly stated that improving mental health of medical staff can significantly reduce turnover intention [33]. What is more, A Chinese study from Wuhan [34] has shown that job satisfaction is an important factor affecting medical staff’s turnover intention, so job satisfaction of the primary medical staff as well as their mental health should be a priority.

Multiple linear regression analysis of this study showed strong significance between job burnout and turnover intention. Combined with the results of Pearson correlation analysis, we found there is a positive correlation between turnover intention and job burnout. That is, the higher the job burnout, the stronger turnover intention, which is consistent with a Chinese study [35]. Moreover, research results from China and other countries confirmed the occurrence of job burnout is often accompanied by lack of mental health and decline in job satisfaction.

## Suggestions

Since our research object is from Anhui Province in the central and eastern part of China, we have put forward the following policy recommendations in response to the above identified problems, which can provide reference for the decision-making of Anhui Province's health services and administrative departments.

Our team found that middle-aged medical staff are more willing to leave, so we suggest medical managers need to immediately improve basic medical facilities, provide skilled doctors with matching hardware facilities, and enable them to better exert their professional advantages and enhance their recognition and confidence in their work. They should also encourage young medical staff and let them actively progress and innovate to train the backup talents of the health workforce to stabilize the primary medical staff. Moreover, it is necessary to strengthen communication and collaboration between primary medical institutions, which is conducive to improving the primary medical system.

Aiming at the problem of higher scores for doctors’ turnover intention, we suggest medical managers pay more attention to development status of doctors and discover the difficulties and problems in their work and personal lives, and then strengthen communication and cooperation with foreign hospital managers and introduce advanced human resource management experience.

We found that different PHC departments have different levels of turnover intention in Anhui Province. So, the health management departments should strengthen research to find problems of village-level medical institutions; higher-level primary medical institutions, such as community medical service centers and township hospitals, should increase assistance and technical guidance to village clinics, or have poorly operating village clinics merged to maximize the integration of medical resources. If there is a large staff turnover in the village clinics, it is necessary to innovate incentives and give certain encouragement policies to urban medical staff, encouraging them to stay in village clinics, and then fill the gap in human resources and improve the operating mechanism of village clinics.

There was a positive correlation between job burnout score and turnover intention score. Therefore, medical managers need to provide comfort, subsidies, and necessary psychological assistance to primary medical staff so they can face stressors and appreciate positive factors in current work. Additionally, Herzberg's two-factor theory [36] cautions managers: the key to improving staff motivation is to invest in incentives, because for the excellent medical staff, endogenous incentives are more attractive than exogenous one’s force. In addition to paying attention to health factors such as compensation and benefits, managers need to determine motivating factors such as career development and job significance, sense of achievement, and challenges, to further retain high-quality medical personnel and stabilize the team of primary health staff.

### Study limitations

Despite the size and findings of the study, it has limitations. First, the collected samples mainly come from central China's Anhui province, but samples from other provinces and other countries should be included and further researched. Secondly, we only analyzed the turnover intention and influencing factors of some primary medical staff in Anhui, limiting the sample size to a specific region. Finally, although many factors affecting the turnover intention are controlled here, there are still potential confounding factors, such as the hardware facilities of medical institutions, regional customs and culture (Additional file 1).

## Conclusions

According to the scoring criteria for the turnover intention, this study found that primary medical staff in Anhui province has a high level of turnover intention. Middle-aged doctors are more likely to have turnover intention than nurses and doctors of other age group. Staff of village clinics have significantly higher turnover intention than those of other primary medical institutions, indicating this province’s primary medical system is likely to remain incomplete, and the village medical units are facing greater development difficulties. Job burnout also profoundly affects the level of turnover intention, which shows that the psychological status of primary medical staff needs to be improved urgently. Medical managers need to think about more than just physical incentives. These findings will provide ideas and inspiration for the management of primary medical staff.

## Supplementary Information

**Additional file 1.** Questionnaire for this study.

## Data Availability

All data generated or analyzed during this study are included in the article published here and its supplementary information files.

## Abbreviations

ANOVA: Analysis of variance
WHO: World Health Organization
CHC: Community Health Service Centers
CHS: Community Health Service Stations
PCQ-24: Psychological Capital Scale
PSSS: Perceived Social Support Scale
CMBI: Chinese Maslach Burnout Inventory
MBI: Maslach Burnout Inventory
SD: Standard deviation
M: Means
GPs: General practitioners
