# Supplementary material for: Current status and related factors of turnover intention of primary medical staff in Anhui Province, China: a cross-sectional study
Source: Hum Resour Health. 2021 Feb 27;19:23. doi: 10.1186/s12960-021-00563-6 (PMC7910782; doi:10.1186/s12960-021-00563-6)
Supplement: Supplementary file 1 — Additional file 1. Questionnaire for this study. [file 12960_2021_563_MOESM1_ESM.docx]

**Survey Questionnaire of Primary Medical Staff in Anhui Province**

*Dear primary medical workers:*

*This is a social survey and research questionnaire from the School of Health Management of Anhui Medical University. Our main purpose is to understand the current working status and related needs of primary medical staff in Anhui Province. Please fill in some information according to your actual situation and views. In addition, the content of this survey will be kept strictly confidential for you. Thank you for your support and cooperation!*

**1. Your basic information**

|  | **Questions and options** |
| --- | --- |
| **A1** | Your gender:①male ②female |
| **A2** | Your age: |
| **A3** | Your Professional titles: |
| **A4** | Your Education level:①secondary school and below ②Associate degree ③Bachelor degree and above |
| **A5** | Your working years: |
| **A6** | Marital status:①married ②unmarried |
| **A7** | Your monthly income(CNY):① <3000 ②≥3000 |
| **A8** | Your occupation:①physician ②pharmacist ③nurse ④medical manger |
| **A9** | Your region: |
| **A10** | Your work unit:①CHC ②CHS ③township hospital ④village clinics ⑤outpatient department |

**2.** **Chinese Maslach Burnout Inventory (CMBI)**

According to the degree of conformity with the actual situation in your personal work according to the following description, please mark "√" on the corresponding figures, "①" means "completely inconsistent", "⑤" means "fully in line", from ① to ⑤ The degree of compliance is from high to low.

|  | **Items** | **Options** |
| --- | --- | --- |
| **B1** | I am very tired |  |
| **B2** | I don't care about the feelings of the work object |  |
| **B3** | I can effectively solve the problem of work |  |
| **B4** | I worry that work will affect my mood |  |
| **B5** | My workmates often complain about me |  |
| **B6** | I can influence others effectively through my work |  |

|  | **Items** | **Options** |
| --- | --- | --- |
| **B7** | I often feel exhausted |  |
| **B8** | I work with cynicism |  |
| **B9** | I can create a relaxed and lively working atmosphere |  |
| **B10** | At the end of the day's work, I feel extremely tired |  |
| **B11** | I often blame my work partners |  |
| **B12** | After solving the problem of the working object. I am very excited |  |
| **B13** | Recently, I was a little depressed |  |
| **B14** | I often refuse requests from people who work |  |
| **B15** | I completed a lot of meaningful tasks |  |

**3.Psychological capital scale (PCQ-24)**

Here are some sentences that describe how you might think of yourself at the moment. There is no right or wrong answer, you don’t need to spend too much time thinking about it, just answer it based on your first feeling. Please judge the degree of conformity of each statement with your own situation and mark "√" in the corresponding column ①=strongly disagree ②=disagree ③=a little disagree ④=a little agree ⑤=agree

|  | **Items** | **Options** |
| --- | --- | --- |
| **C1** | I believe I can analyze long-term problems and find solutions |  |
| **C2** | During the meeting, I was very confident in stating things within the scope of my job |  |
| **C3** | I believe I have contributed to the discussion of hospital development |  |
| **C4** | Within my scope of work, I believe I can help set goals/purposes |  |
| **C5** | I believe I can contact people outside the department and discuss issues |  |
| **C6** | I believe I can present information to a group of colleagues |  |
| **C7** | If I find myself in trouble at work, I can think of many ways to get rid of it |  |
| **C8** | Currently, I am energetically fulfilling my work goals |  |
| **C9** | There are many solutions to any problem |  |
| **C10** | Right now, I think I am successful in my work |  |
| **C11** | I can think of many ways to achieve my current work goals |  |
| **C12** | Currently, I am achieving the work goal I set for myself |  |
| **C13** | When I encounter setbacks at work, I always recover from it quickly and move |  |

|  | **Items** | **Options** | |
| --- | --- | --- | --- |
| **C14** | At work, I will solve the problems I encounter anyway |  | |
| **C15** | If you have to do it at work, you can say that I can also fight independently |  | |
| **C16** | I'm usually calm about the stress at work |  | |
| **C17** | Because I have experienced a lot of hardships in the past, I can now survive the difficult times at work |  | |
| **C18** | In my current job, I feel like I can handle many things at the same time |  | |
| **C19** | At work, when I encounter uncertain things, I usually look forward to the best results |  |  |
| **C20** | Regarding the occurrence of unfavorable things at work, it is considered temporary and can be resolved |  |  |
| **C21** | For my work, I always see the bright side of things |  |  |
| **C22** | I am optimistic about what will happen to my job in the future |  |  |
| **C23** | In my current job, things are going as I want them to |  |  |
| **C24** | At work, I always believe that "behind the darkness is the light, do not be pessimistic |  |  |

**4.** **Turnover intention scale**

Please click "Yes" in the corresponding column, depending on the actual situation of your last month. (1) - Never (2) - Very little (3) - Occasional (4) - Often

|  | **Items** | **Options** |
| --- | --- | --- |
| **D1** | Do you considering quitting your current job? |  |
| **D2** | Do you want to find other jobs of the same nature？ |  |
| **D3** | Do you want to find other jobs of a different nature？ |  |
| **D4** | Based on your current situation and conditions, what are your chances of finding the right position at another agency? |  |
| **D5** | If you know that another agency now has a vacancy that suits you, how likely are you to get the job? |  |
| **D6** | My friends can really help me. |  |

**5.Perceived Social Support Scale (PSSS)**

Please click "Yes" in the corresponding column, depending on the actual situation of your last month. ①Very disagree②very disagree ③slightly disagree ④neutral ⑤agree ⑥very agreeable ⑦very agreeable.

|  | **Items** | **Options** |
| --- | --- | --- |
| **E1** | People (leaders, relatives, co-workers) will be by my side when I have a problem | \| ① \| ② \| ③ \| ④ \| ⑤ \| ⑥ \| ⑦ \| \| --- \| --- \| --- \| --- \| --- \| --- \| --- \| |
| **E2** | I can share happiness and sadness with some people (leaders, relatives, colleagues) | \| ① \| ② \| ③ \| ④ \| ⑤ \| ⑥ \| ⑦ \| \| --- \| --- \| --- \| --- \| --- \| --- \| --- \| |
| **E3** | My family can help me. | \| ① \| ② \| ③ \| ④ \| ⑤ \| ⑥ \| ⑦ \| \| --- \| --- \| --- \| --- \| --- \| --- \| --- \| |
| **E4** | I can get emotional help and support from my family when I need it. | \| ① \| ② \| ③ \| ④ \| ⑤ \| ⑥ \| ⑦ \| \| --- \| --- \| --- \| --- \| --- \| --- \| --- \| |
| **E5** | Some people (leaders, relatives, colleagues) are the real source of comfort when I am in trouble. | \| ① \| ② \| ③ \| ④ \| ⑤ \| ⑥ \| ⑦ \| \| --- \| --- \| --- \| --- \| --- \| --- \| --- \| |
| **E6** | My friends can really help me. | \| ① \| ② \| ③ \| ④ \| ⑤ \| ⑥ \| ⑦ \| \| --- \| --- \| --- \| --- \| --- \| --- \| --- \| |
| **E7** | I can rely on my friends when there are difficulties. | \| ① \| ② \| ③ \| ④ \| ⑤ \| ⑥ \| ⑦ \| \| --- \| --- \| --- \| --- \| --- \| --- \| --- \| |
| **E8** | I can talk to my family about my problems. | \| ① \| ② \| ③ \| ④ \| ⑤ \| ⑥ \| ⑦ \| \| --- \| --- \| --- \| --- \| --- \| --- \| --- \| |
| **E9** | My friends can share my happiness and sorrow with me | \| ① \| ② \| ③ \| ④ \| ⑤ \| ⑥ \| ⑦ \| \| --- \| --- \| --- \| --- \| --- \| --- \| --- \| |
| **E10** | Some people in my life (leaders, relatives, colleagues) care about my feelings. | \| ① \| ② \| ③ \| ④ \| ⑤ \| ⑥ \| ⑦ \| \| --- \| --- \| --- \| --- \| --- \| --- \| --- \| |
| **E11** | My family is willing to help me make decisions. | \| ① \| ② \| ③ \| ④ \| ⑤ \| ⑥ \| ⑦ \| \| --- \| --- \| --- \| --- \| --- \| --- \| --- \| |
| **E12** | I can discuss my problems with my friends. | \| ① \| ② \| ③ \| ④ \| ⑤ \| ⑥ \| ⑦ \| \| --- \| --- \| --- \| --- \| --- \| --- \| --- \| |
